# Uncovering the multi-level response of *Glycine max* L. to the application of allelopathic biostimulant from *Levisticum officinale* Koch

**DOI:** 10.1038/s41598-021-94774-5

**Published:** 2021-07-28

**Authors:** Agnieszka Szparaga, Sławomir Kocira, Pavol Findura, Ireneusz Kapusta, Grzegorz Zaguła, Michał Świeca

**Affiliations:** 1 Department of Agrobiotechnology, Koszalin University of Technology, 75-620 Koszalin, Poland; 2 Department of Machinery Exploitation and Management of Production Processes, University of Life Sciences in Lublin, 20-950 Lublin, Poland; 3 Department of Machines and Production Biosystems, Slovak University of Agriculture in Nitra, Nitra, 949 76 Slovakia; 4 Department of Food Technology and Human Nutrition, College of Natural Science, University of Rzeszow, 35-601 Rzeszow, Poland; 5 Department of Bioenergetics and Food Analysis, Faculty of Biology and Agriculture, College of Natural Sciences, University of Rzeszow, 35-601 Rzeszow, Poland; 6 Department of Biochemistry and Food Chemistry, University of Life Sciences, 20-704 Lublin, Poland

**Keywords:** Plant breeding, Plant development, Plant ecology, Plant physiology

## Abstract

The interest expressed by the agriculture in the category of innovative biostimulants is due to the intensive search for natural preparations. Our study is the first ever to report a complex approach to the use of allelopathic extracts from *Levisticum officinale* Koch. roots in soybean cultivation, includes analyses of morphological observations, and analyses of biochemical indicators. Hot method of aqueous extraction was applied. The extracts were administered via foliar application and soil treatment. Lovage extracts had high contents of polyphenolic compounds and rich micro- and macroelemental composition. The infusions did not contain gibberellic acid and indole-3-acetic acid but the abscisic acid and saccharose, glucose, and fructose were found. The extracts modified soybean plant physiology, as manifested by changes in biometric traits. Plants responded positively by increased yield. Seeds from the treated plants had higher contents of micro- and macroelements, as well as total concentrations of lipids (with a slight decrease in protein content). In addition, they featured changes in their amino acid profile and fatty acid composition. The application of allelopathic biostimulant caused increased concentrations of isoflavones and saponins. The natural biostimulants from *Levisticum officinale* may become a valuable tool in the sustainable agriculture.

## Introduction

The allelopathy evaluates the effect of chemicals produced by plants on the growth and development of other plants^1^. Nevertheless, the allelopathic effects are today classified as biotic stresses. According to Lichtenthaler^2^, however, the stress signaling in plants and their response to stress factors are diversified and associated with various forms of signal reception and transduction in a plant and its organs^3^.

The allelopathy phenomenon itself is a type of an innovative approach used in the agronomic practice that may offer multiple solutions in the context of a diminishing availability of food and a growing global population. By being specialized biochemical factories, the plants produce various active compounds. These compounds—called allelochemicals—can either stimulate or inhibit plant germination and growth, while their application entails low contents of phytotoxic residues in water or soil^4,5^. Therefore, they can serve as substitutes for synthetic plant regulators, which is consistent with a new EU regulation 2019/1009 of the European Parliament and of the Council of 5 June 2019 stating that products based on allelochemicals can be classified as natural biostimulants. The biostimulants represent a group of products, which activate plant growth when used in small doses. These ecosystem-friendly, natural formulations promote plant growth, nutrient uptake, and plant tolerance of stresses. The interest expressed by the contemporary agriculture in this category of bioproducts is due to the intensive search for novel preparations based on natural substances that could effectively replace synthetic agrochemicals^6^. Because the allelochemicals cause no residual nor toxic effects, they are the perfect base components of innovative biostimulants^7^. Thus, the major aim of the allelopathic research is firstly to observe the effects of such preparations and, afterward, to exploit them in agricultural production. Only this approach will allow reducing or eliminating agri-chemicals as well as putting effective methods for the sustainable development of agricultural production and ecological systems into practice^4,8–10^. Thus, research efforts in this area should focus on the search for and study of potentially allelopathic plant species producing more allelochemicals. In addition, initiatives should be undertaken to obtain such forms of preparations and methods of their application that would promote plant growth and crop yield, taking into account mechanisms of their action and their biochemical composition. This may be the right direction in the long run, and following this direction will allow achieving sustainable development of agriculture, environmental and food safety, as well as the important economic stability for farmers^11–16^.

Our earlier results indicated that the extracts produced from *Artemisia absinthium* can be successfully used as effective allelopathic biostimulant in soybean cultivation. The application of such bioproduct increased soybean yield, while their positive effect can be attributed to their phenolic compounds as well as micro- and macroelements. However further research is needed to thoroughly characterize the chemical composition and the mechanisms of action of the allelopathic biostimulants. One of the solutions to these challenges may be seen also in prototyping medicinal plants, which is the first step to create biostimulants of new generation. This approach will not only bring benefits for the natural environment, but will also represent some kind of a designed agronomic tool for ensuring optimal yield and economic profits^17^.

*Levisticum officinale* Koch., growing wild in most parts of the world, including Asia and Europe, can be indicated as a plant exhibiting the allelopathic potential^18^. It belongs to the family Apiaceae, which includes many culinary and medicinal plants that contain multiple compounds exerting broad and varied biological effects^19^. For this reason, lovage can be a candidate for producing an allelochemical biostimulant. However, such a biostimulant cannot be tested only under laboratory conditions. Its field trials should be performed with crops especially sensitive to abiotic stresses, like e.g. soybean and bean. Considering the wide applicability of soybean seeds in the food industry, the greatest challenges seem to be yield quality control and modelling. Hence, the use of an allelochemical preparation in the form of extracts from *Levisticum officinale* can be perceived as an agronomic practice useful in modelling soybean yield by the plant’s secondary metabolism. By this means, it represents a novel, unconventional, and environmentally-friendly approach coupling allelopathy principles and biostimulant use that not only increases crop yield but also enhances phytochemical biosynthesis in plant response to the biotic stress. Obviously, plant response to an allelopathic biostimulant will depend on many factors, including plant physiological condition as well as the dose and application method of the preparation^20^. However, the innovative character of our research lies not only in the development of a novel biostimulating formulation but also in its dualistic nature. This formulation will also have features of an allelopathic preparation, i.e., it will be a ‘multi-component balanced system of biologically-active substances of metabolic origin based on plant raw materials exhibiting a broad spectrum of bio-activities’^21^.

Our study includes analyses of the allelopathic potential of extracts from *Levisticum officinale*, meaningful morphological observations, and analyses of basic metabolic activities and biochemical indicators of soybean plants’ defense systems. We have advanced a hypothesis that the allelopathic biostimulant produced from roots of *Levisticum officinale* would promote the growth and seed yield of soybean (*Glycine max* L.) due to the mechanisms based on the improved key physiological and biochemical process. This assumption was based on the fact that the biotic allelopathic stress is multi-dimensional and can induce various responses at different levels, including biochemical, physiological, morphological, and—ultimately—ecological one in plants treated with allelochemicals.

## Results and discussion

### Chemical composition of *Levisticum officinale* water extracts

#### Phenolic compounds identified in *Levisticum officinale* water extracts

The quantitative identification of infusions from *Levisticum officinale* roots revealed fourteen polyphenolic compounds (having various retention times) (Table 1). The major phenolics identified included quercetin 3-*O*-rutinoside (118.54 μg/mL) and equisetumpyrone (58.87 μg/mL of chlorogenic acid, ascribed to its deprotonated pseudomolecular ion [M-H] at m/z 423 and its fragments at m/z 173 and 161, respectively, at the absorption maximum of 255–352 nm). The water extracts were also rich in chlorogenic acid (39.28 μg/mL) and neochlorogenic acid (16.17 μg/mL).

**Table 1:** Individual and total phenolic compounds identified by UPLC-PDA-MS/MS in *Levisticum officinale* water extracts (average ± SD).

Compound	Rt	λ_max_	[M-H] m/z		Polyphenols content (μg/mL)
	min	nm	MS	MS/MS	Infusion
Neochlorogenic acid	2.30	298sh, 328	353	191, 179	16.17 ± 0.11
Chlorogenic acid	2.97	298sh, 325	353	191, 179	39.28 ± 0.34
Cryptochlorogenic acid	3.05	298sh, 325	353	191, 135	6.86 ± 0.26
Quercetin 3-*O*-glucuronide-pentoside	3.57	259, 350	609	433, 301	2.47 ± 0.60
Quercetin 3-*O*-glucosyl-rhamnoside	3.67	257, 350	609	301	0.86 ± 0.03
Quercetin 3-*O*-glucosyl-rhamnoside	3.76	257, 340	609	301	3.50 ± 0.11
Quercetin 3-*O*-pentoside	3.89	257, 338	433	301	0.90 ± 0.02
Feruloyl-qunic acid	4.04	298sh, 326	367	193, 134	2.44 ± 0.06
Quercetin 3-*O*-glucosyl-rhamnosyl-(p-coumaroyl)hexoside	4.41	255, 314	917	771, 609, 301	0.86 ± 0.01
Quercetin 3-*O*-rutinoside	4.52	255, 352	609	301	118.54 ± 0.03
Equisetumpyrone	4.63	253, 272sh, 371	2 M [842]	423, 173, 161	58.87 ± 0.37
Kaempferol 3-*O*-rutinoside	5.02	264, 340	593	285	4.00 ± 0.01
di-caffeoyl-quinic acid	5.16	298sh, 326	515	353, 191	1.80 ± 0.01
Kaempferol 3-O-rhamnosyl-pentoside	5.33	266, 334	563	285	3.13 ± 0.02
Total					259.68 ± 0.06

The extracts contained also four quercetin derivatives, identified based on deprotonated pseudomolecular ions [M-H] with m/z 301 and showing UV absorption maxima at 255–352 nm. The UPLC-PDA-MS/MS analysis of *Levisticum officinale* root extract revealed also the presence of two quercetin derivates (quercetin 3-*O*-glucuronide-pentoside and quercetin 3-*O*-glucosyl-rhamnosyl-(p-coumaroyl)hexoside)), that were ascribed to the deprotonated pseudomolecular ion [M-H] at m/z 433 and 771, respectively, and to their fragments at m/z 301 and 609. Their UV absorption maximum fitted in the following ranges 259–350 nm and 255–314 nm, respectively. The listed in Table 1 compounds with identical spectral characteristic, measured as a mass to charge ratio (m/z) at 609 indicates the presence of constitutional isomers. As a result of fragmentation patterns, these compounds break down and release a radical ion at m/z 301. This ion indicates the presence in the aglycone part the quercetin—flavonol, very common in nature polyphenolic compound.

The study results demonstrate high concentrations of phenolic compounds in the extracts examined, reaching 259.68 μg/mL on average, that could support natural strategies of soybean plants for coping with adverse stress factors. Besides, such a composition of the extract (i.e., a high concentration of allelochemicals) can increase the availability of nutrients for plants, because—by being the so-called chelating agents—phenolic compounds indirectly contribute to the increased solubility of nutrients in the soil^22^. Even though the higher concentrations of secondary metabolites (also in the form of phenolic compounds) may increase plant resistance, the production of secondary metabolites itself assumes some costs borne by the plants^23–27^. The precise identification of the costs of allelopathy borne by plants remains controversial and disputable^26,28,29^. Scientists have been inconclusive in this regard mainly due to a lack of experimental research addressing this issue. Results of sparse studies depend on the bio-system examined and cannot be extrapolated^30,31^. Nevertheless, phenolic compounds can be found in all plant organs and, therefore, protect it against sun radiation and attacks of pathogens and herbivores, which justifies the use of polyphenols-rich extracts in allelopathic preparations. Their presence in extracts from *Levisticum officinale* induces some type of biotic stress in soybean plants, thereby contributing to their protection against abiotic stress conditions, including particularly oxidative stress^32^. It needs to be emphasized that the production and removal of reactive oxygen species in a cell play an essential role in allelopathic effects. After the administration of allelochemicals, the acceptor plants can rapidly synthesize reactive oxygen species at the contact site with the allelopathic preparation^33,34^, and modify activities of such antioxidative enzymes as superoxide dismutase and peroxidase^35,36^, or ascorbic acid peroxidase^37^. Therefore, this composition of the *Levisticum officinale* extract contributes directly to counteracting oxidative stress. As explained by Shearer et al.^38^, the allelopathic interactions can lead to changes in signal transduction and to the imbalance between the production of reactive oxygen species and antioxidative capabilities of acceptor plants. According to Van Oosten et al.^39^, soybean plants have stress-sensitive phenotypes. The phenotype of these plants can be changed due to the exogenous use of various biostimulants, especially those showing antioxidant and free radical scavenging properties, as well as the ability to chelate metals^40^. Thus, foliar application of *Levisticum officinale* extracts, rich in polyphenolic compounds and micro- and macroelements, could cause their rapid uptake by the leaves and increased accumulation in meristematic tissues. According to Park et al.^41^, the uptake of bioactive compounds in the most sensitive plant tissues has a great importance in cases of thermal and oxidative stress, to which crops are exposed. Exogenous use of allelopathic extracts with compatible solutes such as polyphenols or micro- and macroelements can affect the flow of ions across plant membranes^42^. However, it should be emphasized that the cause-and-effect relationship between the application of biostimulants, phenotypic evaluation of the treated plants and the accumulation of solutes and protection against stress is still not fully understood^43^. According to Nephali et al.^44^ even more careful research doesn't always show a clear answer. It turns out that changes on the transcriptome or proteome levels do not always correspond to changes in biochemical phenotypes. Additionally, they may not fully reflect the exact biochemical condition of plants in response to stress and the application of biostimulants. It should be mentioned that the suggested resistance of plants to oxidative stress, resulting from the composition of the applied allelopathic biostimulants, could also be the result of gene activation, e.g. the *ALDH7* gene, associated with oxidative stress (protective function for plants)^45^. Research by Wang et al.^46^ showed the existence of 51 co-functional networks of *GmALDH* genes along with 1146 co-functional connections that are involved in the processes of lipid metabolism, photosynthesis, proline catabolism and catabolism of small molecules. The application of allelopathic biostimulants led to changes in the concentration of these components in soybeans, which may probably be the result of shaping the expression of these genes. In addition, the activation of these genes, which support soybean against stress conditions, may affect the resistance of soybean plants to oxidative stress, but without changes in plant phenotypes^45^. Although, in own research it was shown that the number of pods and plant height changed, however, further studies are required to indicate the mechanism responsible for the observed phenotypic changes in soybean plants.

#### Multielemental composition and sugars content of *Levisticum officinale* extracts

Table 2 presents the multielemental composition of water extracts prepared from powdered roots of *Levisticum officinale*. Results obtained provide an important clue, indicating that the extracts were highly diversified in their both macro- and microelemental composition. The infusions were especially rich in macroelements. The allelopathic extract had high concentrations of calcium, potassium, magnesium, and sulfur.

**Table 2:** Multielemental composition of water extracts from *Levisticum officinale* obtained by hot extraction (infusion) (average ± SD).

Element	Extract obtained by boiling (infusion)
**Macroelements (mg/mL)**	
Ca	328.833 ± 5.551
K	395.467 ± 8.615
Mg	73.423 ± 0.127
Na	19.987 ± 0.075
P	30.927 ± 0.085
S	75.433 ± 0.188
**Microelements (mg/mL)**	
Cu	0.0417 ± 0.001
Fe	0.178 ± 0.009
Mn	0.672 ± 0.005
Mo	0.007 ± 0.002
Sr	1.064 ± 0.008
Zn	0.377 ± 0.001
**Toxic metals (mg/mL)**	
As	< LLD
Cd	< LLD
Pb	< LLD
**Sugars (mg/mL)**	
Saccharose	3.560 ± 0.021
Glucose	0.210 ± 0.023
Fructose	0.789 ± 0.032
**Active acidity**	
pH	5.95 ± 0.02

< LLD—below limit of detection.

The extracts obtained from a potentially allelopathic plant contained iron, copper, manganese, zinc, and molybdenum. Interesting outcomes of analyses were achieved in the case of molybdenum and zinc. Their levels are especially controlled in soybean biostimulation not only because of their yield-promoting effect but also because of their influence on the appropriate course process of nodulation and, thus, on nitrogen assimilation. While molybdenum is an essential element of an enzymatic complex—nitrogenase, the yield-promoting effect of zinc is primarily due to the activities of plant hormones (auxins in particular), whose deficiency results in growth inhibition^47^. Our previous research on the use of *Artemisia absinthium* extracts also proved that this plant has allelopathic potential. It was shown that the allelopathic biostimulant produced from this plant influenced the development and yielding of soybean. However, compared to *Levisticum officinale* extracts, it was less rich in macro- and microelements. Particularly significant differences were observed in the levels of calcium, magnesium, phosphorus and sulfur. Nevertheless, both preparations showed a biostimulating effect^17^. Summing up this stage of the present study, it can be concluded that the extracts from *Levisticum officinale* contained a pool of elements essential for the successful growth and development of plants. Plant response to the application of the allelopathic extracts was also associated with the effects of micro- and macroelements on certain vital processes, considering the proven capability of selected elements for stimulating the mechanisms of plant resistance to biotic and abiotic stresses or aiding the uptake of other nutrients. In addition, such a multielemental composition of the extracts from *Levisticum officinale* was proved to even compensate for or alleviate the toxic effects of other elements or induce adaptive responses of plants to adverse abiotic stresses^48^.

The research showed that the *Levisticum officinale* extract was characterized by a low acid pH. The water extracts produced contained three carbohydrates, i.e., saccharose, glucose, and fructose (Table 2). Infusions from *Levisticum officinale* roots were especially rich in saccharose. Nearly three-fold lower concentration was noted for fructose and 17-fold lower for glucose. This carbohydrate composition of the allelopathic extracts affects their bioactivity. According to the latest theory presented by Kumar et al.^49^, the positive and initiating effects of extracts from *Levisticum officinale* can be due to the induction of the so-called ‘sweet resistance’ by sugars, namely plant resistance controlled by saccharide compounds that enhances the defense system^49^. By this means, this theory also assumes that the alternative carbohydrate-rich extracts influence the primary antioxidative system of plants by counteracting stress through ensuring cellular equilibrium between redox reactions. This process triggers beneficial changes in the defense system of plants by initiating and inducing their tolerance of stress^50^. The ‘sweet resistance’ is one of the newest concepts in plant stress physiology, while only a few carbohydrates have so far been classified as growth and development promoters. The results of our previous research on prototyping extracts from *Artemisia absinthium* L. for their biostimulating properties showed that these preparations also contained sugars. However, the concentration of individual of them was lower compared to the extracts from *Levisticum officinale*. However, both allelopathic extracts stimulated the yield potential of soybean in three-year field cultivation^17^. The composition of the tested extracts from *Levisticum officinale* shows they are a complicated system of active compounds, with carbohydrate content being one of their strong points. This is due to the multi-faceted role of carbohydrates in plant growth and development. Following the presented theories, it may be concluded that an appropriate content of sugars in allelopathic extracts would determine their biostimulating activity and plant response at the physiological and biochemical levels.

#### Phytohormones in water extracts from *Levisticum officinale*

The extract from *Levisticum officinale* contains a wide range of phytochemicals. Notwithstanding to the mineral nutrient elements, polyphenols and sugars, growth enhancing hormones have not been found in tested extract. Analysis showed that extract did not contain gibberellic acid and indole-3-acetic acid (Table 3). On the other hand, the *Levisticum officinale* water extract contained the abscisic acid in concentration 0.58 ± 0.05 µg/ml. Plant hormones, like abscisic acid, are signaling phytohormones with different regulatory roles in plant metabolism and adaptation to abiotic stresses^51^. ABA stimulates stomatal closure and minimizes water loss by transpiration^52^. Therefore, exogenous ABA application can have a great interest in plant growth biostimulation^53^. The results of our research indicates that allelopathic extract from *Levisticum officinale* elicit the response of soybean plants not only due to the presence of phenolic compounds and multielemental composition but also to ABA concentration. According to Mahdavi et al.^54^ and Zhang et al.^55^ the application of abscisic acid both under optimal and sub-optimal growth conditions can increase water potential and improve chlorophyll concentration in soy bean. Nowadays the role of ABA on the reduction of stress in protein-rich crops has received considerable attention.

**Table 3:** Phytohormones in water extracts from *Levisticum officinale* obtained by boiling (infusion) (average ± SD).

Phytohormone class	Rt	[M-H]^−^	MRM transition		*Levisticum officinale* infusion
			Quality transition	Collision energy	
	min	m/z	m/z	eV	µg/ml
Gibberellic acid GA_3_	4.65	345	347 > 239	35	< LLD
Indole-3-acetic acid IAA	5.59	174	174 > 130	10	< LLD
Abscisic acid ABA	6.71	263	263 > 153	10	0.58 ± 0.05

< LLD—below limit of detection.

### Response of soybean plants to biostimulating allelopathic extracts

#### Soybean yield and biometric traits

The spraying of soybean plants with an extract from *Levisticum officinale* promoted their growth, leading to plant height increase by 13% compared to the control combination (Table 4). The application of the allelopathic extracts into the soybean rhizosphere also improved plant growth, but only by 7% compared to the control combination. The study results showed also that the response of soybean plants to the application of extracts did not affect the first pod location. The foliar application of the allelopathic extract from *Levisticum officinale* increased pod number by 23%, whereas its application into the soil—by 17%, compared to the control combination.

**Table 4:** Response of soybean plants to biostimulating allelopathic extracts—soybean yield and its elements (mean values of 2018–2020 ± SD).

Year	Biostimulant treatment	Plant height, cm	Location height of the first pod, cm	1,000 seeds weight, g	Number of pods, per m^−2^	Seeds yield, g m^−2^
Average 2018–2020	Infusion spraying	121.6 ± 2.2a	11.8 ± 2.1a	169.46 ± 2.91b	1618 ± 164a	409.3 ± 7.0a
	Infusion watering	114.6 ± 3.3ab	13.1 ± 2.0a	162.07 ± 3.93c	1543 ± 181a	383.9 ± 12.5a
	Control	107.6 ± 4.8b	13.2 ± 1.6a	176.82 ± 1.69a	1319 ± 51a	333.3 ± 8.5b

Means in the columns, concerning the selected traits, followed by different letters are significantly different at *p* < *0.05.*

The response of plants to the biotic stress induced by the allelopathic biostimulant was negative, as evidenced by the 1000 seed weight which decreased significantly in the combinations involving plant treatment with the extracts tested. However, this negative plants’ response had no unbeneficial effect on seed yield, which was higher upon the foliar application of the extract from *Levisticum officinale* (by 23% compared to the control combination) (Table 4). The results of our study can be explained by the composition of the allelopathic biostimulants which contained significant amounts of not only polyphenolic compounds but also micro- and macroelements. As reported by Salwa et al.^56^, the microelements of the preparations could have a key influence on crop growth and yield because their availability for plants at particular stages of their phenological development determines the final yield.

However, this response resulted in their increased yield, probably due to the induced activity of their immune system^57,58^, which—in response to the use of allelochemicals—activated many detoxifying enzymes and transporters in order to facilitate toxin inactivation and elimination from plants and to adjust respective metabolic processes^3^.

#### Multielemental composition of seeds from soybean plants treated with extracts from *Levisticum officinale*

Table 5 presents the multielemental composition of seeds of soybean treated with infusions from *Levisticum officinale* roots. The results of analyses demonstrate that the seed yield was highly diversified in terms of macro- and microelemental composition. Inducing the biotic stress in plants through the application of the allelochemical extracts increased concentrations of macroelements in seeds compared to the control treatment. A small decrease was only observed for sulfur concentration.

**Table 5:** Effect of soybean treatment with extracts from *Levisticum officinale* on the content of elements in its seeds (means of 2018–2020 ± SD).

Element	Control	Infusion spraying	Infusion watering
**Macroelements, mg/g**			
Ca	2.4181 ± 0.0222c	2.7075 ± 0.0402a	2.6258 ± 0.0029b
K	14.6573 ± 0.1553c	16.1665 ± 0.1477b	16.6782 ± 0.0293a
Mg	2.6016 ± 0.1540b	2.9458 ± 0.0607a	3.0566 ± 0.0274a
Na	< LLD	< LLD	< LLD
P	7.5424 ± 0.0115c	7.9107 ± 0.0113b	8.0866 ± 0.0166a
S	3.3658 ± 0.0029a	3.3100 ± 0.0043b	3.1508 ± 0.0113c
**Microelements mg/g**			
Al	0.0013 ± 0.0004b	0.0069 ± 0.0009a	0.0021 ± 0.0007b
Cr	0.0002 ± 0.0001b	0.0019 ± 0.0007a	0.0002 ± 0.0001b
Cu	0.0045 ± 0.0001c	0.0057 ± 0.0002b	0.0077 ± 0.0002a
Fe	0.0416 ± 0.0015b	0.0990 ± 0.0084a	0.0434 ± 0.0051b
Mn	0.2995 ± 0.0108a	0.2991 ± 0.0094a	0.3134 ± 0.0066a
Mo	0.0026 ± 0.0001b	0.0039 ± 0.0001a	0.0040 ± 0.0001a
Ni	0.0054 ± 0.0001c	0.0071 ± 0.0001a	0.0066 ± 0.0001b
Se	0.0006 ± 0.0003b	0.0004 ± 0.0002b	0.0015 ± 0.0001a
Sr	0.0095 ± 0.0001c	0.0111 ± 0.0001a	0.0105 ± 0.0001b
Zn	0.0466 ± 0.0001b	0.0465 ± 0.0001b	0.0491 ± 0.0001a
**Toxic metals mg/g**			
As	< LLD	< LLD	< LLD
Cd	< LLD	< LLD	< LLD
Pb	< LLD	< LLD	< LLD

< LLD—below limit of detection; Means in the rows, concerning the selected traits, followed by different letters are significantly different at *p* < *0.05.*

Higher, on average, contents of microelements were determined in the seeds from plants treated with the allelopathic extracts from *Levisticum officinale* in the form of infusions.

The study results presented above indicate that, at this stage of evaluation, the activity of extracts from lovage roots allows classifying them to the group of allelopathic biostimulants. Undoubtedly, they increased the effectiveness of nutrient uptake and assimilation, and improved quality traits of soybean yield. However, such properties of the allelopathic extracts could be achieved only when they were administered by spraying.

#### Protein contents, its amino acid and fatty acids composition in seeds from soybean plants treated with extracts from *Levisticum officinale*

Protein contents and its amino acid composition are presented in Table 6. Globulins were the dominant fraction of soybean seeds. The highest content of this fraction was determined for the control seeds, while the foliar application of infusions caused a slight decreased in this fraction by c.a. 4%. Contrary the seed of plants sprayed with infusion were characterized by higher amount of albumins. In fact, changes in globulins fraction were reflected in slightly lower total content of proteins. It should be noted that content and quality of proteins is affected by both genetic (e.g. variety) and breeding factors (e.g. fertilization, stress). An increase in albumin fraction may be due to over-synthesis of anti-antioxidant enzymes and pathogen-related proteins usually observed after application of natural inductors^59^. On the other hand, a slight decrease in globulins, being a main storage fraction in soy-bean, may arise from utilization of energy in development of systemic acquired resistance rather than in production of storage materials.

**Table 6:** Effect of soybean treatment with extracts from *Levisticum officinale* on the protein content, its amino acid profile and fatty acid composition (means of 2018–2020 ± SD).

Compound		Control	Infusion spraying	Infusion watering
Amino acids (mg/g)	Asp	30.2 ± 1.9a	34.8 ± 1.9a	32.4 ± 1.9a
	Thr	11.4 ± 0.8a	13.3 ± 0.9a	12.4 ± 0.7a
	Ser	14.2 ± 1.0a	16.4 ± 1.1a	15.1 ± 1.0a
	Glu	49.0 ± 3.4a	56.6 ± 3.7a	51.8 ± 3.2a
	Pro	15.6 ± 1.1b	19.5 ± 1.3a	18.5 ± 1.1a
	Gly	11.4 ± 0.7b	13.4 ± 0.8a	12.8 ± 0.8ab
	Ala	11.5 ± 0.7b	13.7 ± 0.9a	12.9 ± 0.8ab
	Val	13.0 ± 0.9a	15.2 ± 1.0a	14.8 ± 0.9a
	Ile	11.4 ± 0.7b	13.5 ± 0.8a	13.0 ± 0.7ab
	Leu	19.5 ± 1.4b	23.3 ± 1.3a	22.2 ± 1.4ab
	Tyr	8.6 ± 0.6a	9.9 ± 0.6a	9.6 ± 0.7a
	Phe	13.0 ± 0.9b	15.4 ± 0.9a	14.5 ± 0.9ab
	His	7.4 ± 0.5a	8.58 ± 0.5a	8.2 ± 0.5a
	Lys	17.8 ± 1.1b	20.9 ± 1.2a	20.3 ± 1.2ab
	Arg	18.7 ± 1.3a	21.7 ± 1.3a	20.5 ± 1.1a
Protein (mg/g)	Total	367.2 ± 4.25a	353.4 ± 5.03b	355.9 ± 2.56b
	Albumins	107.7 ± 3.3b	114.9 ± 3.5a	107.3 ± 3.6b
	Globulins	211.7 ± 5.7a	195.6 ± 3.4c	203.1 ± 1.5b
	Prolamins	21.9 ± 0.4b	20.5 ± 0.3c	23.7 ± 1.3a
	Glutenins	25.9 ± 0.8a	22.4 ± 0.4b	21.8 ± 0.1c
Fatty acids (%)	C14:0	0.09 ± 0.01a	0.09 ± 0.01a	0.11 ± 0.01a
	C15:0	< LOD	0.02 ± 0.00a	0.02 ± 0.00a
	C16:0	11.82 ± 1.19a	11.67 ± 2.06a	11.62 ± 2.01a
	C16:1n7	0.06 ± 0.00c	0.09 ± 0.01b	0.12 ± 0.02a
	C17:0	0.08 ± 0.01ab	0.10 ± 0.01a	0.07 ± 0.01b
	C18:0	3.54 ± 0.36a	3.73 ± 0.35a	3.87 ± 0.65a
	C18:1n9c + C18:1n9t	22.58 ± 3.54a	22.35 ± 1.94a	23.14 ± 4.34a
	C18:2n6c + C18:2n6t	52.41 ± 7.94a	52.54 ± 8.91a	51.69 ± 8.00a
	C18:3n3 (alpha)	6.61 ± 0.52a	6.54 ± 1.14a	6.37 ± 0.94a
	C20:0	0.35 ± 0.03a	0.36 ± 0.04a	0.38 ± 0.07a
	C20:1n9	0.20 ± 0.02a	0.19 ± 0.02a	0.23 ± 0.02a
	C20:2n6	0.03 ± 0.01a	0.03 ± 0.00a	0.03 ± 0.00a
	C21:0	0.02 ± 0.00b	0.02 ± 0.00b	0.03 ± 0.00a
	C20:5n3	< LOD	< LOD	< LOD
	C22:0	0.39 ± 0.04a	0.40 ± 0.04a	0.41 ± 0.04a
	C22:1n9	0.02 ± 0.00b	0.03 ± 0.00a	0.02 ± 0.00b
	C22:2n6	< LOD	0.04 ± 0.00b	0.06 ± 0.01a
	C23:0	0.02 ± 0.00b	0.04 ± 0.00a	0.03 ± 0.01ab
	C24:0	0.11 ± 0.01	< LOD	< LOD
	SFA	16.42 ± 1.75a	16.43 ± 3.02a	16.54 ± 1.91a
	MUFA	22.86 ± 3.01a	22.66 ± 1.58a	23.51 ± 4.20a
	PUFA	59.05 ± 11.08a	59.15 ± 5.71a	58.15 ± 10.46a
	OMEGA 3	6.61 ± 1.00a	6.54 ± 0.55a	6.37 ± 0.83a
	OMEGA 6	52.44 ± 4.94a	52.61 ± 5.47a	51.78 ± 5.97a
	OMEGA 9	22.80 ± 2.53a	22.57 ± 2.01a	23.39 ± 1.72a
Total fat content (g/100 g DM)		19.16 ± 0.58b	22.70 ± 0.71a	22.48 ± 0.64a

Means in the rows, concerning the selected traits, followed by different letters are significantly different at *p* < *0.05.*

The soybean seeds differed also in their amino acid profile. In spite of lower contents of total proteins the seeds obtained after treatments were characterized by increased amounts of aminoacids. It may be explained by the fact that the control seeds contain higher amount of globulins that in soy-beans are built with up to 40% of sugar moieties^60^. Significant changes were noted in contents of all amino acids, presumably due to the effect of the plant extracts on this metabolic pathway in plants. In turn, proline content was observed to increase. This amino acid can be found in various plants, including especially those exposed to stress conditions. It has been reported to serve many physiological functions, like osmoregulation or energy and nitrogen absorption. It has also been shown to serve as an ageing signaler and the so-called stress sensor^61^.

The application of *Levisticum officinale* extracts also induced arginine accumulation. It needs to be emphasized that poor transamination of arginine can lead to amino acid degradation and promote ammonia production^62–64^. The branched-chain amino acids (isoleucine, valine, and leucine) occurred in higher concentrations in the seeds from crops in which the allelopathic extracts were administered through foliar application.

Furthermore, the study results showed varied responses of plants to the treatment with allelopathic extracts manifested in the levels of alanine, aspartic acid, and glutamic acids in their seeds. Higher contents of these amino acids were determined in the seeds from plants treated with the extracts than in the control seeds. In response to the stress factor, these amino acids act as active precursors of other macromolecules because glutamic acid is involved in nitrogen metabolism and TCA cycle, whereas aspartic acid and alanine in the transamination. The differences in the amino acid composition of soybean seeds could be due to the coupled effect of various factors; however contents of amino acids in soybean seeds were not necessarily correlated with the total protein content of the seeds.

The results of the study concerning the fatty acid composition of soybean seeds point to the ambiguous response of the plants to the biotic stress induced by the application of extracts from *Levisticum officinale* (Table 6). The relative percentage of fatty acids differed among soybean seeds depending on the combination tested. Concentrations of some of the fatty acids increased, while those of the others decreased in soybean seed.

However, it needs to be emphasized that the concentration of unsaturated fatty acids in soybean seeds is one of key indicators of their quality, considering their final application and market price. Oleic, linolenic, linoleic, and elaidic acids are regarded as extremely important and valuable unsaturated fatty acids of soybean seeds. The use of extracts from *Levisticum officinale* in the form of spraying resulted in slightly increased concentrations of linoleic and linoleaidic acids, compared to the control seeds. Somehow different observations were made for the levels of oleic and elaidic acids. Their concentrations increased in the seeds from one combination, namely that where the allelopathic extracts were applied by spraying. In the case of α-linolenic acid, soybean treatment with infusions from *Levisticum officinale* decreased its content in the seeds. Soybean plants responded to the treatment with extracts via the enhanced accumulation of unsaturated palmitoleic acid in seeds, compared to the control samples. Interesting dependencies were observed for cis-13,16-docosadienoic acid, which was not detected in the control seeds, but appeared in the seeds from plants treated with *Levisticum officinale* extracts.

The study results show also the effect of various methods of extract application on the levels of saturated fatty acids in soybean seeds. The content of palmitic acid was observed to decrease, whereas contents of stearic and arachidic acids—to increase upon soybean plant treatment with *Levisticum officinale* extracts. In turn, lignoceric acid present in control seeds was not detected in the seeds from plants treated with the allelopathic preparations.

In the case of soybean seeds, important are not only the contents of individual fatty acids but also the ratio of unsaturated to saturated fatty acids. The plant response to the application of infusions from *Levisticum officinale* was manifested by a higher content of saturated fatty acids. In turn, plant watering with the extracts stimulated an increase in the content of monounsaturated fatty acids in the seeds, compared to the control samples. While, contents of polyunsaturated fatty acids increased as a result of the foliar application of the extracts in soybean cultivation.

Soybean plants’ response to the biotic stress was also manifested in changes in the unsaturated to saturated fatty acids ration in seeds. They reached 4.98 and 4.94, compared to 4.99 noted in the control samples. The lower ratios are indicative of the lower percentages of oleic, linoleic, and linoleic, and higher percentages of palmitic and stearic acids synthesized in soybean seeds. An increased percentage of unsaturated fatty acids compared to the saturated fatty acids shows that plant treatment with the extracts had a small negative impact on the nutritional value of soybean seeds.

The increased contents of unsaturated fatty acids in soybean seeds can be ascribed to the effect of extracts from *Levisticum officinale* on the enhancement of biosynthesis and desaturation enzymes of the fatty acid metabolic pathway. This beneficial effect could be due to the contents of macro- and microelements because certain microelements, like iron, are essential for the synthesis of ferredoxin, which plays an important role in NADPH as an electron donor to stearyl desaturase in the higher plants^65^.

The application of extracts modified also soybean response expressed in the levels of omega 3, 6, and 9 acids. In the case of omega 3 acids, plant treatment with infusions contributed to a slight decrease in their content in the seeds, compared to the control treatment. The level of omega 6 acids increased in the seeds from plants treated with the extracts by spraying. In turn, the content of omega 9 acids tended to increase, compared to the control, only upon extracts application into the soil.

#### Isoflavonoid and saponins compounds identified in seeds from soybean plants treated with extracts from *Levisticum officinale*

Plant response to the biotic stress, manifested in the accumulation of daidzin, genistin, and glycitin isoflavones, and their corresponding conjugated forms is presented in Table 7. The results of our study demonstrate that the application of allelopathic biostimulants in soybean cultivation caused changes in the contents of individual isoflavones. The differences observed were due to the various methods of biostimulant application. The total concentration of these bioactive compounds in seeds increased after plant treatment with infusions in the form of spraying and watering (an increase by over 55% and 36%, respectively, compared to the control samples). The assessment of soybean plant response to the use of allelopathic biostimulants revealed that they responded to this type of biotic stress with an increased concentration of individual isoflavones in seeds.

**Table 7:** Individual isoflavonoid and saponins compounds identified by UPLC-PDA-MS/MS in soybean seeds (means of 2018–2020 ± SD).

Individual isoflavonoid (µg/g)							
Compound	Rt	λ_max_	[M-H] m/z		Control	Infusion spraying	Infusion watering
	min	nm	MS	MS/MS			
Daidzin	4.21	248	417	255	11.52 ± 0.72c	18.54 ± 1.06a	14.07 ± 0.82b
Glycitin	4.39	257	447	285	5.30 ± 0.23c	9.70 ± 0.40a	8.17 ± 0.43b
Genistin	5.16	259	433	271	13.47 ± 0.78c	22.28 ± 0.89a	19.01 ± 0.71b
Malonyldaidzin	5	257	503	417, 255	38.16 ± 3.27c	53.05 ± 0.44a	45.58 ± 0.43b
Malonylglycitin	5.38	250	533	447, 271	8.82 ± 0.39c	15.28 ± 0.31a	13.41 ± 0.39b
Glycytein	5.42	257sh, 319	285	239	4.81 ± 0.18b	7.84 ± 0.61a	6.89 ± 0.45a
Malonylgenistin	6.24	258	519	433, 271	44.55 ± 2.79b	70.20 ± 3.85a	65.58 ± 0.42a
Daidzein	6.76	257	255	207	4.75 ± 0.32c	7.65 ± 0.28a	6.57 ± 0.38b
Acetyl genistin	7.13	260	475	433, 271	1.48 ± 0.16b	2.08 ± 0.31a	1.96 ± 0.12ab
Genistein	8.09	260	271	132	2.23 ± 0.22b	3.14 ± 0.32a	2.84 ± 0.13a
**Total**					**135.08 ± 8.99c**	**209.75 ± 3.34a**	**184.09 ± 0.10b**

Individual saponins (μg/g DM)							
Compound	Rt [min]	[M-H]^−^	Control	Infusion spraying	Infusion watering		
Soyasaponin I	4.01	941.5	468.32 ± 7.59b	651.36 ± 26.90a	487.98 ± 19.35b		
Soyasaponin II	4.13	911.5	172.32 ± 7.46b	196.00 ± 5.41a	182.99 ± 2.44ab		
Soyasaponin III	4.15	795.4	181.81 ± 2.97a	167.82 ± 0.41b	172.55 ± 2.95ab		
Soyasaponin A_2_	3.66	1105.5	241.34 ± 5.03b	265.69 ± 7.35a	234.58 ± 2.44b		
Soyasaponin A_1_	3.53	1267.5	217.38 ± 9.08ab	237.91 ± 3.45a	213.01 ± 2.15b		
Soyasaponin IV	4.23	765.4	169.15 ± 9.35a	191.93 ± 2.75a	179.99 ± 44.67a		
Acetyl-Soyasaponin A_1_	4.35	1435.6	84.43 ± 1.19c	99.44 ± 0.91b	121.73 ± 4.10a		
Acetyl-Soyasaponin A_2_	4.23	1273.5	267.17 ± 1.50b	331.21 ± 8.62a	262.37 ± 7.95b		
Acetyl-Soyasaponin A_3_	4.32	1243.5	58.84 ± 3.30b	76.90 ± 0.74a	56.62 ± 1.75b		
Soyasaponin A_3_	3.7	1075.5	215.32 ± 10.67b	280.32 ± 0.76a	200.19 ± 4.7b		
Soyasaponin A_4_	3.94	1237.5	141.50 ± 5.85a	150.07 ± 5.04a	143.82 ± 2.8a		
Acetyl-Soyasaponin A_4_	3.56	1363.6	37.30 ± 0.31b	37.94 ± 0.15b	38.94 ± 0.35a		
Acetyl-Soyasaponin A_5_	3.7	1201.5	41.57 ± 0.79a	43.85 ± 0.98a	42.47 ± 0.31a		
Acetyl-Soyasaponin A_6_	3.36	1171.5	33.00 ± 0.81b	51.58 ± 1.12a	29.62 ± 0.47b		
(Acetyl)-Soyasaponin A_c_	4.35	1419.6	47.42 ± 3.91b	59.14 ± 1.43a	51.55 ± 1.91ab		
(Acetyl)-Soyasaponin A_d_	4.26	1405.6	42.30 ± 1.14b	49.17 ± 1.96a	40.14 ± 0.67b		
(Acetyl)-Soyasaponin V	3.57	957.5	119.31 ± 13.24a	131.46 ± 6.43a	121.23 ± 1.57a		
SoyasaponinBd	4.26	939.4	137.27 ± 13.80b	167.42 ± 0.95a	136.98 ± 6.62b		
Soyasaponin Be	3.78	955.4	86.30 ± 8.70b	106.70 ± 0.72a	86.24 ± 2.41b		
Soyasaponin BdA	4.11	1083.5	34.79 ± 1.04b	60.52 ± 0.38a	55.83 ± 1.62b		
Soyasaponin BeA	3.89	1067.5	16.59 ± 0.80b	24.17 ± 0.30a	16.50 ± 0.14b		
Soyasaponin βa	4.43	1037.5	16.33 ± 0.17b	22.69 ± 0.18a	16.63 ± 0.41b		
Soyasaponin γg	4.54	921.4	74.17 ± 3.85b	93.77 ± 0.98a	76.55 ± 1.44b		
Soyasaponin γa	4.61	891.4	37.00 ± 1.86b	55.94 ± 0.06a	62.76 ± 1.29a		
**Total**			**2940.93 ± 7.52b**	**3553.01 ± 29.20a**	**3031.28 ± 9.92b**		

Means in the rows, concerning the selected traits, followed by different letters are significantly different at *p* < *0.05.*

The analyses demonstrated malonylglucosides (malonylgenistin and malonyldaidzin), followed by genistin to be the major forms of isoflavones in soybean seeds. In turn, the lowest content was determined for acetyl genistin.

According to Kim et al.^66^, the acetyl forms are only products of degradation formed from malonate. In these authors’ opinion, it is also likely that all isoflavonoids accumulated in the seeds were earlier in the form of malonyl. The results of our study demonstrate that the application of extracts from *Levisticum officinale* influenced the synthesis of isoflavonoids, probably because soybean seeds were the main site of their synthesis. However, the synthesis process itself was initiated by active compounds of the allelopathic biostimulant. The literature indicates that some pool of isoflavonoids in seeds can result from their transport from other morphological parts of the plants^66,67^. Nevertheless, as emphasized by Kim et al.^66^, the accumulation of flavone compounds in soybean seeds is also largely dependent on the cultivar, meteorological conditions, and plant exposure to stress factors at the stage of seed growth and development^66,68,69^.

Flavonoids represent a large class of plant secondary metabolites, exhibiting antioxidative effects in the higher plants, which are exposed to or challenged with various stress-inducing factors^70–72^. This hypothesis may explain changes in their concentration in soybean seeds caused by the extracts from *Levisticum officinale*. Biotic stress induction in plants by the allelochemicals could affect the activities of enzymes involved in the oxidation process of polyphenols^73^. At this stage of the study, we may also conclude that the preparation tested revealed the traits of a biostimulant because it influenced the metabolic pathways in soybean plants. Plant response to this type of stress was manifested in the beneficial change in the concentrations of biologically-active compounds in the seeds. This positive response could be due to the fact that the plants exposed to severe stress conditions accumulate flavonoids substituted by a dihydroxy-B-ring that are effective scavengers of reactive oxygen species^74^.

The analyzed soybean seeds contained many types of saponins, classified to group A, B, and E (Table 7). The application of the allelopathic water extracts in the form of plant spraying and watering contributed to an increased content of saponins in the seeds. In soybean seeds tested, we detected the presence of soyasaponin I, II, III, and IV. The highest concentration was reported for soyasaponin I. Soybean seeds contained also saponin BeA, which is a saccharide, 2,3-dihydro-2,5-dihydroxy-6-methyl-4H-pyran-4-one (DDMP), attached through an acetal linkage to the C-22 hydroxyl of algycones of soyasaponin I. Results of previous investigations^75,76^ also show that soyasaponins I, II, III, and IV are saponin forms conjugated with DDMP.

The analysis of soybean seed composition revealed additionally 4 types of group A saponins, called A1, A2, A3, and A4 (having retention times of 3.53, 3.66, 3.70, and 3.94 min, respectively). The highest concentration was determined for soyasaponin A3. This group of saponins was also affected by plant treatment with the allelopathic extracts, which resulted in their increased levels in the seeds.

Among the acetyl-soyasaponins A, the largest group in soybean seeds was represented by acetyl-soyasaponins A1. Results of our analyses demonstrate also the presence of B group saponins (7 groups) containing DDMP, called soyasaponins Bd, Be, BdA, BeA as well as βa, γg, and γa. The most abundant group in soybean seeds was represented by soyasaponins Bd, whereas the smallest one by soyasaponins βa. Results of our study demonstrate that the use of extracts from *Levisticum officinale*, in the form of both foliar application and soil treatment, increased the content of this group of saponins in soybean seeds.

Our study also showed that the application of extracts from lovage affected the isoflavone to soyasaponin ratio. Plant response to this type of biotic stress was manifested in its value increase from 0.046 in the control samples to 0.059 in the seeds from plants sprayed with the extract; with the maximal reported value at 0.061. However, according to Kim et al.^66^, changes in the contents of flavonoids and saponins are not correlated. Nevertheless, the most important finding from our study is that the application of the completely natural, allelopathic biostimulant offers an agronomic tool enabling to increase contents of compounds, whose bio-activity determines the health-promoting values of soybean products^66^.

It is noteworthy that the increased levels of saponins determined in soybean seeds are due to complicated reactions proceeding in plants also under the influence of stress factors, mainly as a consequence of modified gene expression. As reported by Shimoyamada et al.^77^, the Sg-3 gene encodes glucosyltransferase, which afterward catalyzes the glucosylation of the galactosyl group of saponin A. In turn, Tsukamoto et al.^78^ have proved that the Sg-4 gene is an arabinosyltransferase which catalyzes the arabinosylation of glucuronic acid residue attached to C-3 of soyapapolyols. However, the above authors have emphasized that these glycosyltransferases have not been identified so far. A study conducted by Kurosawa et al.^79^ supported these observations, as these authors have demonstrated that soybean glucuronosyltransferase is a specific enzyme of the UDP-glucuronic acid as a donor and of soyapogenols as an acceptor. In addition, Kurosawa et al.^79^ confirmed that this enzyme played an important role in the biosynthesis of saccharide chains in soyasaponins. In turn, Shibuya et al.^80^ characterized two soybean glycosyltransferases, i.e. *GmSGT2* and *GmSGT3*. An essential conclusion from their study is that *GmSGT3* transfers the rhamnosyl group from UDP-rhamnose not only to soyasaponin III but also to soyasaponin γa. In addition, studies have shown that the application of the allelopathic extract affected the biosynthesis of the main soyasaponin in seed, i.e. Soyasaponin. According to the Shibuya et al.^80^ this compound is biosynthesized by the sequential addition of glucuronic acid, galactose, and rhamnose to soyasapogenol B. The results of research conducted by these authors, indicate that UDP-galactose (*GmSGT2*) and UDP-rhamnose (*GmSGT3*) are involved in the biosynthesis of soyasaponin I in soybean. Therefore, further research on allelopathic biostimulators is necessary, showing their influence on the activity of genes involved in the biosynthesis of these bioactive compounds in *Glycine max*. Although still scarce information is available on the molecular mechanisms underlying the glycosylation process, which play the key role in soyasaponin biosynthesis^81,82^. The results obtained in our study allow us to conclude that the allelopathic formulations induced the metabolic response of plants, manifested by changes in the quality and quantity of saponins in the seeds.

The exogenous application of extracts from *Levisticum officinale* triggered varied responses of soybean plants, depending on the method of their administration. Its extracts had high contents of polyphenolic compounds and rich micro- and macroelemental composition. The application of the allelopathic extracts modified soybean plant physiology, as manifested by changes in their biometric traits. Besides, soybean plants responded positively to this type of biostimulant by increased yield. The allelochemicals contained in the extracts modified the basic metabolic activities and biochemical indicators of soybean. Seeds obtained from the treated plants had higher contents of individual micro- and macroelements, as well as total concentrations of lipids (with a slight decrease in protein content). In addition, they featured changes in their amino acid profile and fatty acid composition. Analysis showed that extract did not contain growth regulators such as gibberellic acid and indole-3-acetic acid but the abscisic acid was found. The most important outcome of this study includes the increased contents of bioactive compounds, as the seeds produced by plants treated with the allelopathic biostimulant showed significantly increased concentrations of isoflavones and saponins. Considering the multi-level response of soybean plants, the foliar application of the analyzed allelopathic extracts is strongly recommended.

All observations made over the three-year study performed in real field conditions enable concluding that soybean plants responded positively to the biotic stress induced by the allelopathic biostimulant.

To recapitulate, the natural biostimulants based on allelopathic extracts from *Levisticum officinale* may become a valuable tool in both the sustainable and organic agriculture. However, our research needs to be continued to fully exploit and elucidate their effects. The genetic approach would seem indispensable to identify the key enzymes and genes involved in plant response, which will, in turn, pave the way to the agricultural applications of these allelopathic biostimulants.

## Methods

All methods were carried out in accordance with relevant guidelines and regulations.

### Plant material and growth conditions

Soybean seeds of Abelina variety (purchased from the producer of the eligible seed—Saatbau Polska Sp. z o.o., https://www.saatbau.com/pl) were sown during a three years field experiment (2018–2020) conducted in Perespa (50º66’N; 23º63'E, Poland). The experiment was designed and performed in a random block system in four replications, on experimental plots with the size of 15 m^2^. Plants were grown on soil classified as Gleyic Phaeozems (pH in 1 M KCl 7.3–7.4). The average level of available nutrients in 100 g of soil was as follows: 12.7–14.1 mg P_2_O, 15.1–17.2 mg K_2_O, 6.4–6.9 mg Mg and 8.0–9.4 mg N–NO_3_ + N–NH_4_. *Triticum aestivum* L. was used as the previous crop. Seeds were sown on the 2 May 2018, 2019, and 2020 with 4.0 cm gaps in rows with 30 cm spacing. In each growing season, plants were treated with the infusions from *Levisticum officinale*. Extract was applied in the form of double plant spraying (300 L·ha^−1^) or double soil treatment (600 L·ha^−1^) at the BBCH 13–15 (trifoliolate on 2nd or 3rd node unfolded) and BBCH 61 (10% flowers open—beginning of flowering) developmental stages of soybean (BBCH—Biologische Bundesanstalt, Bundessortenamt and Chemical Industry). Combinations with plants sprayed and watered with water used for extract preparation served as the control. Spraying was performed with the Pilmet 412 LUX (Unia, Grudziądz, Poland) sprayer equipped with nozzles air-induction flat fan nozzles 6MSC (working pressure 0.30 MPa). The soil application was made using Hose drop system for boom sprayers (Agroplast, Sawin, Poland). After the pods have matured, when the seeds have obtained a typical color and hardness (BBCH 89—full maturity: approx. all pods are ripe; soybeans final colour, dry and hard), plants were harvested. Determinations were conducted for plant height, location height of the first pod, number of pods, 1,000 seed weight. Dates of application were chosen based on results of our earlier experiments addressing the use of natural and synthetic biostimulants in soybean cultivation^83^.

### Extract production

Extracts were prepared from dried, ground roots of *Levisticum officinale*. The dried root was purchased from the producer Runo Poland, certified (PL-EKO-07–04,901) on the basis of Article 29 (1) of Regulation (EC) No 834/2007 and of Regulation (EC) No 889/2008. The declared operator has submitted his activities under control, and meets the requirements laid down in the named Regulations. The purchased herbal material has the status of a certified product No. PL-EKO-07–0490 (18) document No. Z 001 17 from 16/08/2017 (http://runobio.pl/wp-content/uploads/2018/02/certyfikat-RUNO-BIOEKSPERT-Sp.-z-o.o.-2017.pdf). Infusions from lovage were prepared by the hot extraction method, i.e. 5 g of *Levisticum officinale* were added to 250 mL of distilled water. The solution was boiled in a water bath for 30 min. Then the solution was left in a dark place for 48 h, at the temperature 4 °C. Afterwards, extracts were centrifuged at 4250 rpm for 5 min and filtered through a blotted filter paper Whatman no. 1^84^. An active acidity (pH) was measured as well using a VOLTCRAFT KBM-110 m (Conrad Electronic SE, Hirschau, Germany) with a pH electrode.

### Chemical composition of infusions from *Levisticum officinale*

Extracts from roots of *Levisticum officinale,* were determined for their chemical composition. Their contents of elements and sugars as well as composition and contents of phenolic compounds and phytohormones concentration were analyzed. Determination of mineral content in extract was assessed using the method of Zaguła et al.^85^ by ICP-OES analysis. Identification and quantification of polyphenols in extract was studied exactly as previously described by Oszmiański et al.^86^. by the UPLC-PDA-MS method. Sugars in extracts from *Levisticum officinale* were evaluated based on the EN 12,630, 1999 standard and procedure of Pereira da Costa and Conte-Junior^87^, using the HPLC system. The phytohormones analysis in extracts from *Levisticum officinale* was assessed according to the procedure of Šimura et al.^88^, by the UPLC-PDA-TQD with an ESI method.

### Plant yielding and seeds chemical composition determination

The seed yield and fat concentration in soybean seeds were determined. Total fat content was analyzed based on the acid hydrolysis method^89^. Determination of fatty acids in soybean seeds following the methods of Zhang et al.^90^, using gas chromatograph. Protein sequential fractionation was done using the procedure described by^91^. The protein fractions content was determined according to Bradford^92^ using a calibration curve of albumins, globulins, prolamins, and glutenins of beans as standard protein. Hydrolysis of protein into amino acids has been carried out according to Davies and Thomas^93^, using HPLC analysis. Extraction and purification procedure for determination of isoflavones and saponins in soybean seeds was determined following the procedures of Kapusta et al.^94^. The isoflavones were identified by an UPLC Aquity system consisting of binary solvent manager, sample manager, column manager, PDA detector and tandem quadrupole mass spectrometer (TQD) with electrospray ionization mode (ESI)^95^. Saponins in soybean seeds were qualified and quantitated using the same system mentioned above, based on the method described by Jervis et al.^96^. The measurements of microelements and macroelements in soybean seeds were performed by ICP-OES (Inductively Coupled Plasma Optical Emission Spectrometers, Thermo iCAP Dual 6500, USA)^85^.

### Statistical analysis

In statistical analyses, the number of replications for each combination in each study year was N = 12. The mean result from twelve replications was the average value for the each year of the experiment. The evaluation of the normal distribution of data was performed using Shapiro–Wilk test. The obtained results were analyzed using the one-way ANOVA. The estimation of significance of differences between mean values (comparison between methods of soybean plants treatment) based on Tukey confidence intervals, at a significance level of *p* < *0.05*. Due to the number of recorded results, the article presents the average results from three years of field experiments (results presented in tables, with the standard deviation ± SD) Statistica 13.3 software (TIBCO Software Inc., USA) was used for analyses of the results.

### Ethics approval

The experimental research and field studies on plants, including the collection of plant material, complied with relevant institutional, national, and international guidelines and legislation. Soybean cultivation and harvesting were carried out in accordance with the European Coexistence Bureau (ECoB) Best Practice Document for the coexistence of genetically modified soybean crops with conventional and organic farming^97^.
